# Use of direct and iterative solvers for estimation of SNP effects in genome-wide selection

**DOI:** 10.1590/S1415-47572010005000014

**Published:** 2010-03-01

**Authors:** Eduardo da Cruz Gouveia Pimentel, Mehdi Sargolzaei, Henner Simianer, Flávio Schramm Schenkel, Zengting Liu, Luiz Alberto Fries, Sandra Aidar de Queiroz

**Affiliations:** 1 Animal Breeding and Genetics Group, Department of Animal Science, Georg-August-University of Göttingen, GöttingenGermany; 2 Departamento de Zootecnia, Faculdade de Ciências Agrárias e Veterinárias, Universidade Estadual Paulista Júlio de Mesquita Filho', Jaboticabal, SPBrazil; 3 Department of Animal and Poultry Science, Centre for Genetic Improvement of Livestock, University of Guelph, Guelph, ONCanada; 4 Vereinigte Informationssysteme Tierhaltung w.V., Verden / AllerGermany; 5 GenSys Consultores Associados S/S Ltda., Porto Alegre, RSBrazil

**Keywords:** breeding value, genomic selection, mixed model equations, numerical method

## Abstract

The aim of this study was to compare iterative and direct solvers for estimation of marker effects in genomic selection. One iterative and two direct methods were used: Gauss-Seidel with Residual Update, Cholesky Decomposition and Gentleman-Givens rotations. For resembling different scenarios with respect to number of markers and of genotyped animals, a simulated data set divided into 25 subsets was used. Number of markers ranged from 1,200 to 5,925 and number of animals ranged from 1,200 to 5,865. Methods were also applied to real data comprising 3081 individuals genotyped for 45181 SNPs. Results from simulated data showed that the iterative solver was substantially faster than direct methods for larger numbers of markers. Use of a direct solver may allow for computing (co)variances of SNP effects. When applied to real data, performance of the iterative method varied substantially, depending on the level of ill-conditioning of the coefficient matrix. From results with real data, Gentleman-Givens rotations would be the method of choice in this particular application as it provided an exact solution within a fairly reasonable time frame (less than two hours). It would indeed be the preferred method whenever computer resources allow its use.

## Introduction

Most applications in animal breeding involve the solution of systems of equations with very large numbers of unknowns. For instance, with multiple or single trait animal models or random regression test-day models the number of parameters increases as a function of the number of animals being evaluated, which are not so rarely in the hundreds of thousands or above. The coefficient matrices that arise from these kinds of problems are too large to be stored in high speed memory. For this reason, iterative solvers, both on data or on the mixed model equations, have gained high popularity and are widely employed in genetic evaluation of livestock. When an iterative algorithm is applied for the solution of a linear system, one cannot obtain the elements of the inverse of the coefficient matrix. Therefore, prediction error variances and standard errors of estimates have to be calculated in an indirect way and are consequently approximated values.

Recent advances in molecular genetic techniques have led to the availability of information on a large number of sequence variations across the genome. This new source of information has launched a series of studies (*e.g.*, Meuwissen *et al.*, 2001; Xu, 2003) attempting to associate such sequence variations with phenotypic variation in complex traits. If the number of identified variations is large enough that it covers the entire genome one could assume that most of the quantitative trait loci (QTL) associated with a given trait will be in linkage disequilibrium with at least some of these markers. Genome assisted breeding values (GEBV) could then be calculated by estimating the effects of QTL associated with the markers, or alternatively the effects of the markers themselves, on the traits of interest and taking the summation of these effects across the whole genome. High-density panels for genotyping thousands of single nucleotide polymorphisms (SNP) are now commercially available and their costs are likely to decrease over time, which would make genome-wide selection an affordable procedure in the near future (Schaeffer, 2006). The number of unknowns in the models considered for the estimation of SNP effect on total genetic merit of individuals is a function of the number of genotyped SNP, which may be constant over time. A constant number of unknowns and the increasing rate of advances in computer hardware could make the use of a direct solver an interesting option, as that would make it possible to obtain the elements of the inverse of the coefficient matrix for calculating standard errors of estimates.

This research compares the application of iterative and direct solvers for the estimation of SNP effects with potential use on genomic selection. Possible computational advantages and disadvantages of the methods were investigated.

## Material and Methods

###  Simulated data

A simulated data set prepared for the XII QTL-MAS Workshop, held in Uppsala, Sweden, in May 2008, was used. Data consisted of phenotypes, breeding values and genotypes for 6,000 SNP on 5,865 animals. Markers were evenly distributed on six chromosomes of 100 cM each. Details about the simulation procedures can be found in Lund *et al.* (2009).

From the 6,000 markers, 75 had the same genotype shared by all animals and were then discarded from the analyses. Twenty five subsets were extracted from the data set, resembling scenarios that differed in the number of evenly spaced markers (1,200, 2,400, 3,600, 4,800 and 5,925) and in the number of genotyped animals (1,200, 2,400, 3,600, 4,800 and 5,865).

###  Real data

Relative performances of the compared numerical methods were also investigated with their application to real data. A set of 3081 Holstein-Friesian sires genotyped for the Illumina BovineSNP50 BeadChip was used. Following usual data quality control procedures, markers with a call rate (*i.e.*, successfully assigned genotype) lower than 90% or minor allele frequency lower than 1% were excluded from the initial set. After filtering, 45,181 markers were kept in the analyses. The response variable used was the estimated breeding value for milk yield.

###  Equations to solve

A multiple linear regression model (Xu, 2003) was employed to estimate SNP effects on breeding values. The model equation is described below:


(1)
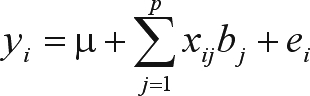


where *y*_*i*_ is the breeding value of the *i*^th^ animal; μ is an overall mean; *x*_*ij*_ is the coefficient for the *j*^th^ SNP genotype of the *i*^th^ animal; *b*_*j*_ is the slope on the *j*^th^ SNP genotype; *p* is the number of genotyped SNP; *e*_*i*_ is a random error.

Coefficients *x*_*ij*_ were set to -1 for genotype A_1_A_1_, 0 for genotype A_1_A_2_ and +1 for genotype A_2_A_2_. Regression coefficients can be obtained by the solution of the following set of mixed model equations:


(2)
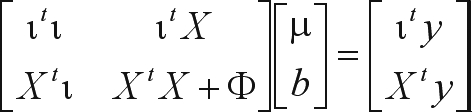


where ι is a vector of ones, of order equal to the number of genotyped individuals; Φ is a square matrix of order *p*.

Different statistical models can be defined by alternate ways of setting up the matrix Φ. For the sake of simplicity, and as the purpose of this investigation was to compare numerical rather than statistical methods, the matrix Φ used in the application to the simulated data was assumed to be an identity matrix.

In the application to the real data, a different procedure was conducted in order to resemble a more realistic scenario. The matrix Φ used was an identity matrix times a ratio of variances, as in the ‘BLUP' method by Meuwissen *et al.* (2001). Furthermore, two alternate forms of ‘BLUP' analysis were performed: one in which the set of equations to solve and matrix Φ were as previously described and another in which each observation was weighted by the effective number of daughter contributions, a measure of the reliability of the corresponding estimated breeding value, which represents a procedure more likely to be used in practice. The second set of equations was of the form:



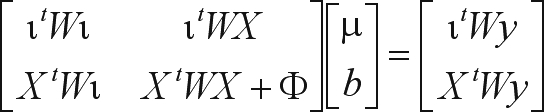


where *W* was a diagonal matrix with *w*_*ii*_ element equal to the effective number of daughter contributions (Fikse and Banos, 2001) of sire *i*.

###  Numerical methods

One iterative method and two direct (decompositional) methods were used in this study. The iterative method of choice was Gauss-Seidel with Residual Update (GSRU), which is a variant of Gauss-Seidel iteration due to Janss and de Jong (1999). The modification from Gauss-Seidel consists in updating the vector of residuals in each iteration. A thorough explanation of the method including the pseudo-code in Fortran 95 (which was used here) is provided by Legarra and Misztal (2008) in a study of possible computational methods for use in genome-wide selection. The convergence criterion employed here was the relative difference between two consecutive solutions (Lidauer *et al.*, 1999) and the stopping point was defined when this difference was lower than 10E-14.

One of the direct methods considered was Cholesky Decomposition (CHD), which was also included in the study by Legarra and Misztal (2008). Briefly, it consists in a factorization of a positive-definite coefficient matrix into the product LL^t^ where L is a lower triangular matrix. Therefore the application of the method involves setting up the mixed model equations, factorizing the left hand side and solving two triangular systems.

The other direct method employed in this study was a row-wise algorithm for orthogonal (QR) decomposition, namely Givens planar rotations (Givens, 1954). According to Bai *et al.* (2001), orthogonal factorization is strongly robust and numerically stable. The standard form of Givens rotations requires a square root operation per rotation and four multiplications for each pair of off-diagonal elements in the pivot (*r*) and working (*x*) rows:


(3)
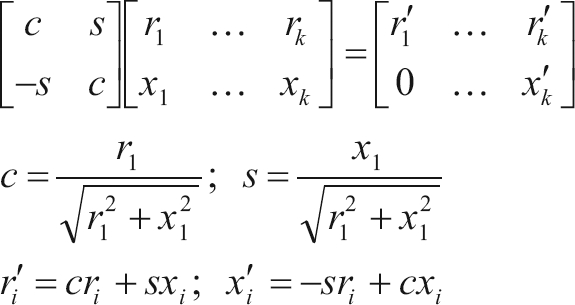


Following the rationale underlying the square root free version of Gram-Schmidt orthogonalization, Gentleman (1973) proposed a modification of the procedure in order to save the square root operation in (3). The modification consists in finding not the triangular matrix R (from the QR decomposition) itself, but rather a diagonal matrix D and a unit upper triangular matrix 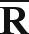 such that:


(4)
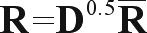


The modified algorithm thus rotates a row of the product 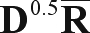 with a scaled row of X:



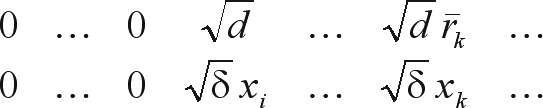


and the transformed rows can be written:



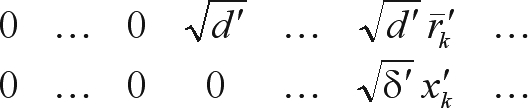


where


(5)
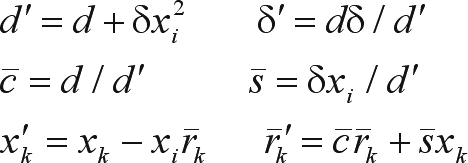


Besides avoiding the square root operation in (3), the updating formulae in (5) also take only three multiplications for each pair of elements involved in the rotation. Actually, with a slight modification, an extra multiplication could still be saved, but it would compromise stability (Bai *et al.*, 2001). Here an algorithm using the formulae in (5) presented by Gentleman (1974) was applied and referred to as Gentleman-Givens (GG) algorithm.

For all the scenarios simulated analyses were run using the three numerical methods, and computational requirements in terms of time and memory were measured. All the analyses of the simulated data were run on a 2.0 GHz Intel® E4400 processor in a PC with 2.0 Gb of RAM. The analyses with the real data required a larger amount of memory and were therefore run on an SGI Altix 4700 system in which each computer blade had a Dual-Core Itanium2 Processor (1.6 GHz clock speed, 533 MHz frontside bus, 16 Mb 3 level cache) with at least 8 Gb of RAM.

## Results

Table 1 presents total processing times and memory requirements for methods GG, GSRU and CHD for the different numbers of animals and markers considered in the simulated data. In all scenarios identical solutions were obtained by the three methods. The iterative method did not have any problem related to convergence and the defined convergence criterion with stopping point at 10E-14 was effectively achieved. The average number of iterations was 582 ± 50.

**Figure 1:**
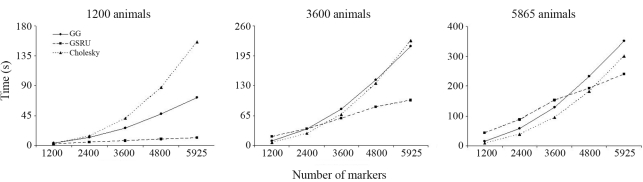
Relative performance of the methods for the lowest, intermediate and the largest number of animals in the simulated data set.

**Figure 2:**
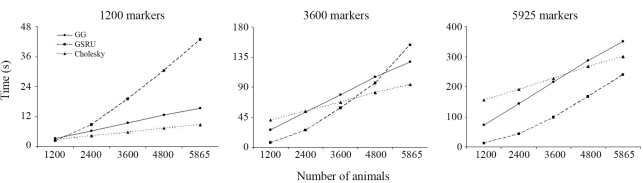
Relative performance of the methods for the lowest, intermediate and the largest number of markers in the simulated data set.

In the situation with the lowest number of markers GSRU was the fastest when the number of animals was also the lowest. As the number of animals increased, processing time with GSRU increased at a higher rate than with GG and CHD. Moreover, as the number of markers increases, the advantage of GSRU over the direct methods persists over an increasing number of animals, up to the point when it is the fastest method for any number of animals. However, in terms of computing time, it is expected that the direct methods finally outperform GSRU when the number of animals becomes very large. This trend is illustrated in Figures 1  and 2.

Regarding the application to real data, the amount of high speed memory (using single precision storage) demanded by the direct methods was 3.8 Gb, whilst GSRU required 530 Mb. Memory requirements obviously did not change between the weighted and non-weighted analyses. Differences in relative performance among methods with respect to time were observed though. When no weight was applied the processing time required for the analyses was 597, 6,235 and 41,011 s for GSRU, GG and CHD respectively. The solutions from GSRU converged (relative difference between two consecutive solutions lower than 10E-14) after 3,513 iterations.

**Figure 3:**
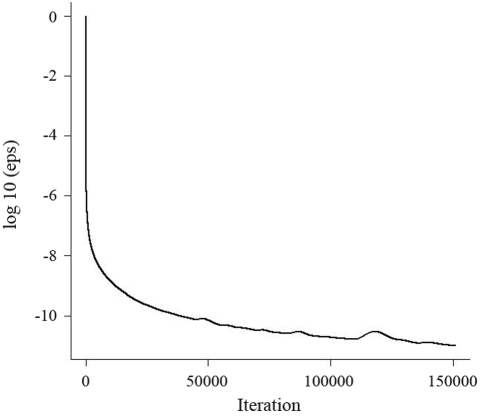
Log10 of relative difference between consecutive solutions across iterations in GSRU applied to the weighted analysis of the real data.

With the weighted version of the analyses the processing time required by the direct methods did not change: 6,228 and 40,856 s for GG and CHD respectively. However, although weighting the observations did not cause any exact linear dependency, it turned the coefficient matrix to be ill-conditioned and considerably slowed down the rate of convergence with GSRU. The log10 of relative differences between consecutive solutions across iterations are shown in Figure 3. A less stringent stopping point (10E-12) had to be set in order to obtain solutions within a reasonable time frame. This threshold was achieved after 150,850 iterations. Solutions obtained at this point were almost the same as the ones obtained with the direct methods. Correlation was greater than 0.999 and mean and maximum absolute difference between SNP effect estimates were 0.07 and 0.72 respectively. The time required to achieve this level of convergence was 24,759 s. An even less stringent stopping point (10E-10) was also tried and solutions converged at this level in 1989 s after 12,073 iterations, but the mean and maximum absolute difference between estimates increased to 0.18 and 1.78 respectively. As pointed out by Legarra and Misztal (2008) it may be possible to speed up the rate of convergence by the use of a relaxation factor, but this factor would have to be determined empirically. Such an approach was not conducted here in order to keep the different performances of the algorithms comparable.

## Discussion

Results from the simulated data illustrate the relative performance of the different numerical methods and their deviations from what would be expected from just a complexity analysis of the algorithms. With fewer animals GSRU was the fastest method regardless the number of markers whilst processing time sharply increased when using a direct solver, more pronouncedly with CHD. Legarra and Misztal (2008) compared several numerical methods to be considered in SNP effect estimation using a data set with ~11,000 SNPs and ~2,000 animals. They fitted a model with additive and dominance effects so the total number of unknowns was ~22,000. In their study, the processing times with GSRU and CHD were 1.5 and 136 min, respectively.

As it can be seen from Table 1, the main advantage of GSRU over GG and CHD is the low memory requirement, which makes it suitable for situations when the number of parameters is very large. The memory requirement of GSRU was a linear function of the number of parameters while memory required by GG and CHD grew quadratically with the number of parameters.

Based on the numbers of arithmetic operations involved in each procedure one would expect that GSRU would be faster than both CHD and GG for increasing number of markers, unless a very large number of iterations were necessary. The number of operations involved in CHD is np^2^/2 for setting up the mixed model equations and (1/3)p^3^ for the factorization, where p is the number of unknowns and n the number of observations. The QR decomposition via GG requires 2np^2^ operations. On the other hand, in GSRU the number of operations is 3np times the number of iterations.

Since GG implies the application of a sequence of planar rotations on the rows of the data matrix, setting up the normal equations is not necessary. One then has to add some pseudo-observations to the system in a way that is equivalent to setting up the equations in (2). The data matrix to be factorized becomes:


(6)
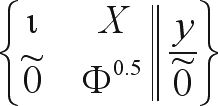


For the diagonal variance structure of the random effect, the above redefinition is fairly straightforward and the elements of the pseudo-observations matrix are easy to compute.

Augmenting the data matrix by a set of p pseudo-observations would dramatically increase the amount of arithmetics needed for decomposition, but this can be avoided with the following procedure. The order by which the rows of data are rotated does not affect the factorization into the product QR. Therefore, the pseudo-observations can be rotated first. Since they consist of just a diagonal matrix this can be achieved by simply initializing this diagonal as the initial diagonal of R (instead of a vector of zeros), which avoids the extra p^2^ transformations implied by the augmentation. This strategy was applied here and made the addition of the pseudo-observations as (almost) costless as the addition of matrix Φ to the random part of the MME in (2). It explains in part why GG was still faster than CHD in situations with larger numbers of markers and fewer animals, as it would be expected based on the number of operations in the case of non-augmented matrices (*e.g.*, fixed regression).

Legarra and Misztal (2008) pointed out that in large SNP data applications setting up the MME incurs in a large amount of non-zeros on the left hand side of the equations, which makes the system fairly dense. Therefore, with CHD it is not possible to take any advantage of the sparsity resulting from the form of parameterization applied here. On the other hand, the use of GG makes it possible to explore sparsity (not in terms of storage but in terms of savings in arithmetic operations). Since the MME are not explicitly set up, all the zeros corresponding to heterozygous genotypes in the data matrix are preserved, and this can be readily exploited by simply skipping the transformation. An example evidence of these savings can be illustrated by the comparison between the processing times required by GG and GSRU in the scenario with the largest number of animals and markers. Based on the number of operations alone (2np^2^ versus 3np times 950 iterations), one would expect that the difference in processing time between GG and GSRU would be almost seven times the observed one. The difference in processing time between GG and CHD when applied to real data was also much larger than would be expected from the number of operations only. It also reflects the impact of these savings in the relative performance of the algorithms.

**Figure 4:**
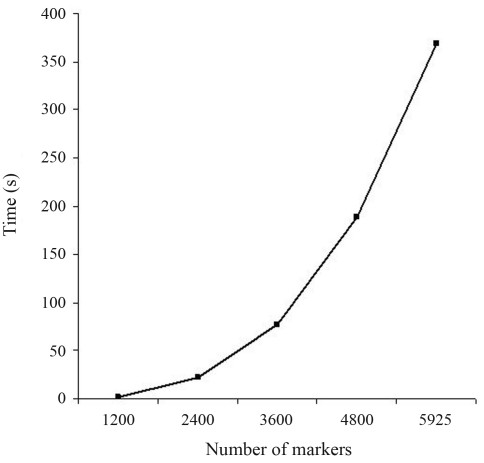
Processing times (in seconds) for estimating standard errors using Gentleman-Givens /Cholesky decomposition, for different numbers of markers in the simulated data.

The use of a direct solver can allow for calculations that are not possible with an iterative approach. For instance, a property of GG is that the upper triangular R obtained by QR factorization of a matrix A is equivalent to the Cholesky factor of the coefficient matrix A^t^A from the normal equations. Given that property, it is possible to calculate the elements of (A^t^A)^-1^ directly from R without the need for explicitly setting up the normal equations. Since A^t^A = R^t^R then (A^t^A)^-1^ = R^-1^R^-t^. To simplify the notation, let's call (A^t^A)^-1^ = M with columns m1, m2, mn, so that RM = R^-t^. Then, since R is upper triangular and R^-t^ is lower triangular, and noticing that the i^th^ diagonal of R^-t^ is the reciprocal of the i^th^ diagonal of R, it is possible to compute the elements of columns mn to m1 from bottom to top, one column at a time. An efficient algorithm for computing the diagonals of the inverse can be found in Lawson and Hanson (1974). Figure 4 presents the time required for calculating exact standard errors of estimates from the simulated data using their algorithm for obtaining the diagonals of the inverse. It can be seen that the increase in time is approximately exponential and therefore this procedure is feasible only in the case of applications with limited number of parameters. For instance, with the real data used in this study the calculation was not even attempted. Because of over-parameterization problems in the genomic selection context, efforts are underway to reduce the dimensionality of the problem (*e.g.*, Long *et al.*, 2007; Habier *et al.*, 2009). In the case of pre-selection of a subset of most relevant markers (*e.g.*, tag SNP) a direct solver could be used for computing (co)variances of SNP effects. In this case, method GG as implemented here could be an interesting alternative to CHD for a few thousand genotyped animals. If a larger panel, say 50 k, is still preferred and one is interested in computing (co)variances, this would be possible by blocking the SNPs of interest. For instance, one would likely be interested in investigating covariances among marker effects within some segments of the genome (*e.g.*, chromosome-wise). Reordering the SNPs of interest to be in the bottom part of R, one can easily compute the inverse of the last portion of the upper-triangular matrix. In the 50 k panel, the largest number of markers within a chromosome was less than 3000, so the computation would take just a few minutes, as shown in Figure 4.

As discussed earlier, with increasing number of markers the difference in speed between the iterative and direct solvers used here tends to become more relevant. Nevertheless, processing time is not the only issue to be addressed in the comparison. In fact, since the matrices to be handled in these applications are dense, memory requirements are major factors in the decision on which method to use. In the implementation of GSRU the data matrix was kept in high speed memory during the whole execution, because the size of problem tested here (both with the simulated and real data) allowed for it. For larger problems though the program can be easily adapted to read it from hard disk, keeping memory requirements at a very low level. On the other hand, the direct solvers required the allocation of p*(p+1)/2 positions of memory in a half-storage structure. For five or ten thousand markers this would mean approximately 190 Mb (in single precision) at most, which is not such a high demand even for current personal computers. The memory requirement increases quadratically with the number of markers. With the application to the real data for example, moving to ~45,000 markers took about 3.8 Gb and required the use of a more powerful machine. Therefore, memory space and computer resources availability are usually the main issue determining the applicability of direct methods.

The relative performance of the solvers may differ in different scenarios, as shown with the simulated data in Figures 1  and 2 and with the real data from the results of the weighted and unweighted analyses. It is therefore difficult to give a general recommendation on which method to use. From the results of the analysis with the real data in the weighted case, GG would be the method of choice in this particular application as it provided an exact solution within a fairly reasonable time frame (less than two hours). It would indeed be the preferred method whenever computer resources allow its use. If the number of markers to be used in the estimation of genomic breeding values were in the hundreds of thousands, then GSRU would definitely be the method of choice, if not the only practically feasible due to memory requirements and the current status of computing power. One would then have to experimentally define a relaxation factor in order to speed up convergence rate. For the current dimension of the SNP effect estimation problem in a farm animal context (*i.e.*, ~50 k) the direct methods were still applicable in practice. It is clear that computer and genotyping technologies are both developing with an outstanding rate of progress so it is reasonable to anticipate that in many future situations a direct solver may be still applicable.

## Figures and Tables

**Table 1:** Computational requirements for solving the equations using Gentleman-Givens (GG), Gauss-Seidel with Residual Update (GSRU) and Cholesky decomposition (CHD), for the different numbers of animals and markers contemplated in the simulated data set.

Number of animals	Number of markers	Method							
		GG			GSRU			CHD	
		Time^1^	Memory^2^		Time	Memory		Time	Memory
1,200	1,200	3.1	2.75		2.3	2.75		2.6	2.75
	2,400	11.8	10.99		4.4	5.49		14.0	10.99
	3,600	26.3	24.73		7.0	8.24		40.4	24.73
	4,800	47.4	43.95		9.3	10.99		87.5	43.95
	5,925	71.9	66.97		11.5	13.56		156.0	66.97

2,400	1,200	6.2	2.75		8.7	5.49		4.2	2.75
	2,400	23.6	10.99		15.4	10.99		20.0	10.99
	3,600	52.6	24.73		25.4	16.48		54.0	24.73
	4,800	94.9	43.95		36.7	21.97		111.2	43.95
	5,925	143.8	66.97		43.4	27.12		191.8	66.97

3,600	1,200	9.4	2.75		19.0	8.24		5.7	2.75
	2,400	35.4	10.99		35.3	16.48		26.0	10.99
	3,600	78.9	24.73		58.9	24.72		67.2	24.73
	4,800	142.3	43.95		83.3	32.96		134.9	43.95
	5,925	215.6	66.97		98.4	40.68		227.8	66.97

4,800	1,200	12.6	2.75		30.4	10.99		7.3	2.75
	2,400	47.2	10.99		53.9	21.97		32.6	10.99
	3,600	105.2	24.73		96.0	32.96		82.1	24.73
	4,800	189.7	43.95		131.6	43.95		161.4	43.95
	5,925	287.5	66.97		167.5	54.24		268.8	66.97

5,865	1,200	15.3	2.75		42.9	13.42		8.7	2.75
	2,400	57.6	10.99		87.3	26.85		38.2	10.99
	3,600	128.2	24.73		153.2	40.27		94.2	24.73
	4,800	232.0	43.95		192.4	53.70		182.9	43.95
	5,925	351.4	66.97		240.2	66.28		301.4	66.97

^1^in seconds. ^2^in Megabytes.
